# NURSES’ PAIN ASSESSMENT AND TREATMENT OUTCOMES IN NURSING HOME RESIDENTS WITH DEMENTIA AND APATHY

**DOI:** 10.1093/geroni/igad104.2753

**Published:** 2023-12-21

**Authors:** Yo-Jen Liao, Ying-Ling Jao, Diane Berish, Marie Boltz

**Affiliations:** Penn State University, State College, Pennsylvania, United States; Pennsylvania State University, State College, Pennsylvania, United States; The Pennsylvania State University, University Park, Pennsylvania, United States; Pennsylvania State University, State College, Pennsylvania, United States

## Abstract

Pain is often undermanaged in nursing home (NH) residents with dementia-related apathy; an understanding of related nursing practices is warranted. This descriptive study examined nurses’ pain assessment and treatment outcomes in NH residents with dementia and apathy. A two-week chart review of nursing progress notes was extracted. Residents’ apathy measured by the Apathy Evaluation Scale (AES) of at least 40 was required. Descriptive and correlation statistical analyses were performed. Seventeen residents were recruited from three NHs; only one NH required daily pain assessment. All residents had pain-related diagnoses (mean=3), with Gastro-esophageal reflux disease and arthritis being the two most common diagnoses. Thirty-four pain-related documentations were identified in progress notes, with 19 pain-related events (e.g., falls, tooth extraction). Among the 19 events, 4 had follow-up documentation of pain management, while the other 15 did not. Among the 34 pain-related documentations, only six described residents’ behaviors, and three used a pain assessment tool (Numeric Rating Scale). More frequent as-needed analgesic administration was associated with lower agitation (-0.56, p< 0.05), yet this association was not found in scheduled analgesic use. Residents who reported pain more frequently received more as-needed analgesics (0.63, p< 0.01). Resident apathy levels were not correlated with analgesic prescriptions. Altogether, nurses recognized residents’ pain reports. As-needed analgesic use correlated with better pain management outcomes; however, pain-related events were not adequately followed up and/or documented. More research is needed to examine the association between nursing pain management practices and resident outcomes in this population.

